# Sarcoidosis with Both Plaques and Subcutaneous Nodules Associated with Combination Therapy with Peginterferon **α**-2a and Ribavirin

**DOI:** 10.1155/2011/741293

**Published:** 2011-09-29

**Authors:** M. Kondo, M. Nishii

**Affiliations:** Department of Dermatology, Ise Municipal General Hospital, 3038 Kusube-cho, Ise, Mie 516-0064, Japan

## Abstract

A 64-year-old woman with hepatitis C was treated using combination therapy with peginterferon **α**-2a and ribavirin. After 3 months, she presented with raised nodules on her knees and elbows. After 8 months, she developed painful subcutaneous nodules on her forearms. We diagnosed sarcoidosis with both plaques and subcutaneous nodules associated with combination therapy with peginterferon **α**-2a and ribavirin. Sarcoidosis with both plaques and subcutaneous nodules is very rare. The patient had a sustained Th2 response in the liver. And a sustained Th1 response occurred only in the skin. It is likely that, for this reason, sarcoidosis was localized to the skin, and the patient developed sarcoidosis with both plaques and subcutaneous nodules.

## 1. Introduction

Combination therapy with interferon (IFN) and ribavirin is the standard treatment for hepatitis C. Both IFN and ribavirin induce a T helper cell (Th) response. Sarcoidosis is a systemic granulomatous disease that is triggered by a Th1 response. IFN-induced sarcoidosis is well documented, and some cases of cutaneous sarcoidosis have been reported [1–4]. In the current study, we report a very rare case of sarcoidosis with both plaque and subcutaneous nodule formation.

## 2. Case Report

A 64-year-old woman with hepatitis C was started on a treatment regimen of ursodeoxycholic acid. Liver enzymes could be normalized, but a negative hepatitis C virus (HCV) ribonucleic acid (RNA) value could not be achieved. Therefore, the patient began combination therapy with peginterferon*α*-2a 180 mg/week and ribavirin 600 mg/day. After 2 months, the peginterferon *α*-2a dose was reduced by 50 percent due to a decrease in neutrophilic leukocytes. The HCV RNA level decreased, and liver enzymes remained normal. Three months after combination therapy was initiated, the patient presented with several asymptomatic, raised, brawny red nodules on the knees and elbows.

**Figure 1:**
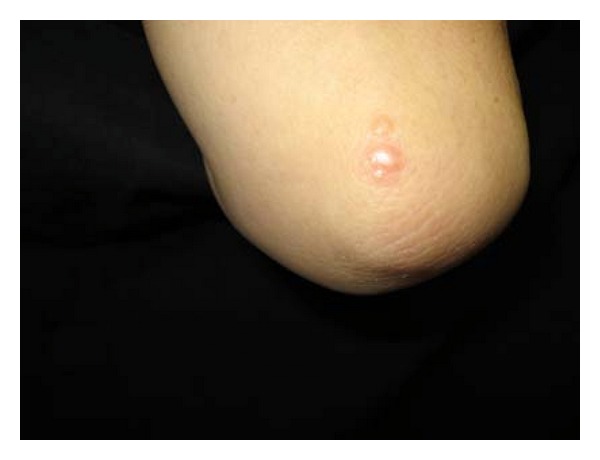
Raised, brawny red nodules of the elbow.

**Figure 2:**
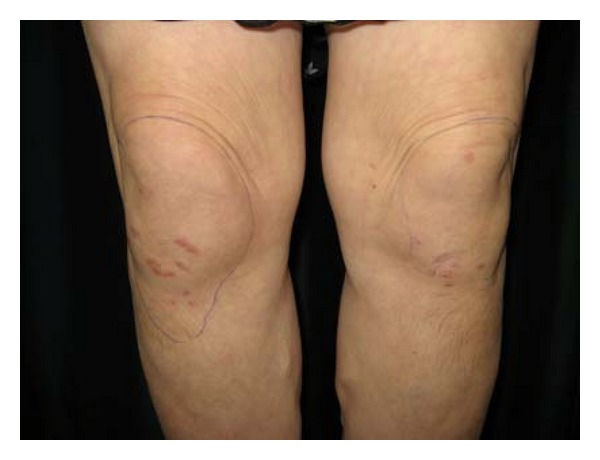
Raised, brawny red nodules of the knee. The outline indicates the range of subcutaneous nodules.

**Figure 3:**
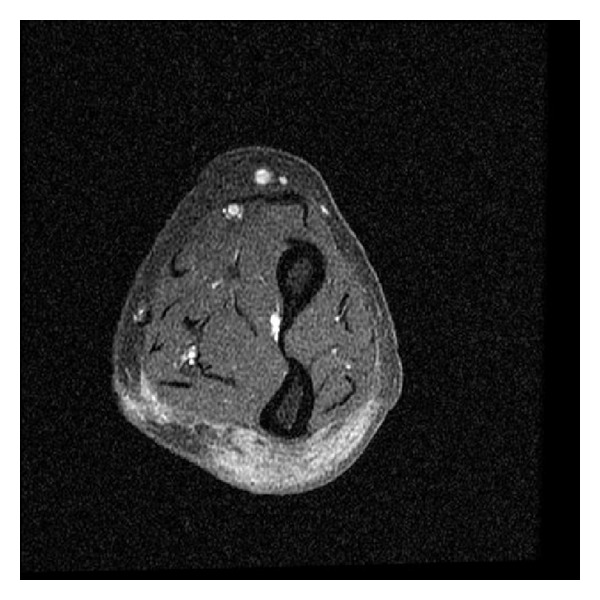
MRI scan (T1-weighted image) of the forearm. The image shows an area of low intensity at the subcutaneous level.

**Figure 4:**
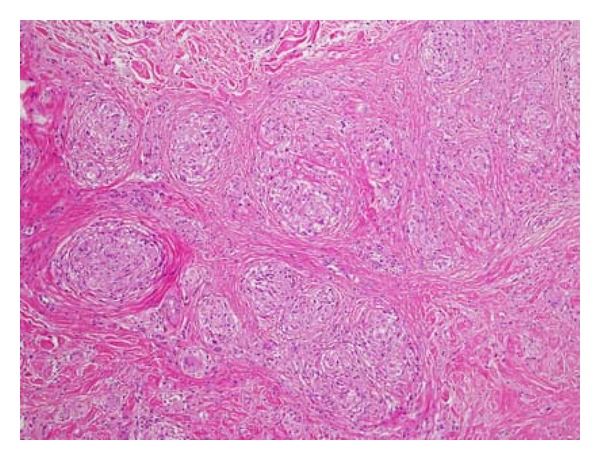
Biopsy of a superficial elbow nodule. Pathologic findings showed evidence of epithelioid granuloma surrounded by a rim of lymphocytes (hematoxylin and eosin stain, ×40).

 At first, the nodules were characterized by disappearances and reappearances. However, 7 months after starting combination therapy, the patient complained of pain in her knees and elbows. Nodules in these regions were clearly visible (Figures 1 and 2). X-ray showed normal soft tissue and bones on the presence of lesions. Eight months after the initiation of combination therapy; however, the patient developed painful subcutaneous nodules on her forearms. A magnetic resonance imaging (MRI) scan (T1-weighted image) showed an area of low intensity suggestive of lymphoma (Figure 3), and a portion of the lesion was excised immediately for diagnostic purposes. Pathologic findings showed epithelioid granuloma, suggesting a diagnosis of sarcoidosis. In addition, a skin biopsy of a superficial elbow nodule was performed. Pathologic findings showed a similar histology to that of the subcutaneous lesion (Figure 4). The skin biopsy also revealed the following laboratory findings: alanine aminotransferase (ALT) 15 IU/L, aspartate aminotransferase (AST) 22 IU/L, alkaline phosphatase (ALP) 165 IU/L, and *γ*-glutamyltransferase (*γ*-GTP) 16 IU/mL. The angiotensin-converting enzyme (ACE) level was slightly elevated at 22.7 U/L, while the lysozyme level was normal at 4.8 *μ*L/mL. A chest X-ray showed no active disease, and the cardiac electrogram showed no abnormality. The patient's eye examination was unremarkable. Based on these findings, a diagnosis of sarcoidosis with both plaques and subcutaneous nodules was made. Since we suspected that sarcoidosis was caused by peginterferon *α*-2a and ribavirin therapy, these drugs were discontinued, and ursodeoxycholic acid therapy was restarted at the original dose. Observation of the superficial and subcutaneous nodules continued, with no additional therapy. Gradually, these nodules disappeared, and the patient was pain free. Four months after stopping the combination therapy, no subcutaneous nodules were found on palpation, and no superficial nodules could be detected with the unaided eye. An MRI scan of the lesions of the knees and elbows showed no abnormality. In addition, the ACE level normalized at 19.7 U/L, and the lysozyme level decreased to 4.2 *μ*L/mL. After several years, the patient has had no reactivation of sarcoidosis, and liver enzymes are maintained within normal limits by the administration of ursodeoxycholic acid.

## 3. Discussion

Sarcoidosis is an autoimmune systemic disease characterized by the Th1 immune response. IFN-*α* is known to stimulate the Th1 cells, and those produce leukotrienes that seem to be responsible for the development of the granulomas. Ribavirin also enhances the Th1 response. Therefore, combination therapy with IFN-*α* and ribavirin is a trigger for sarcoidosis. Many cases of cutaneous sarcoidosis have been reported as a result of combination therapy for hepatitis C [1–4]. However, sarcoidosis with both plaques and subcutaneous nodules is very rare. 

The recognition of sarcoidosis with both plaques and subcutaneous nodules was based on several factors. In the current case, the Th1 response was not enhanced in the liver, but the Th2 response improved. It has been reported that patients with chronic hepatitis demonstrate an enhanced Th2 response [5]. In the case reported here, the sustained Th2 response was due to the long-standing presence of hepatitis C. In addition, it is thought that, in cases of sustained virologic response (SVR), enhancement of the Th1 response in the liver is more common than nonenhancement of the Th1 response [6]; the current patient showed no enhanced Th1 response. Instead, a dose reduction was required due to side effects of combination therapy. Furthermore, the patient's liver was unable to fully metabolize the drugs of the combination regimen. Consequently, the Th1 response in the liver was insufficient. Since there was sufficient blood flow in the liver, Th2 cells were carried by the bloodstream to other organs. However, the skin is far removed from the liver and its microcirculatory blood flow. 

Peginterferon *α*-2a has a long duration of action, with maximum plasma concentration (Tmax) occurring from 72 to 96 hours. Therapeutic blood levels of the drug are sustained for 168 hours. In addition, peginterferon *α*-2a is administered by subcutaneous injection. Thus, the drug was confined to the skin. It is likely that sarcoidosis was localized to the skin for this reason.

## 4. Conclusion

This paper describes a rare case of sarcoidosis with both plaques and subcutaneous nodules. Th2 cells reached other organs through the bloodstream because of the sustained Th2 response in the liver. However, the skin is far removed from the liver. Thus, only the patient's skin showed an enhanced Th1 response.
